# Blue and red tides in the Chesapeake Bay watershed: Examining political and environmental framings of collective action during the 2016 and 2020 elections

**DOI:** 10.1371/journal.pone.0298962

**Published:** 2024-06-21

**Authors:** Stephen Mainzer, Emily L. Pakhtigian

**Affiliations:** 1 Department of Landscape Architecture, The Pennsylvania State University, University Park, Pennsylvania, United States of America; 2 School of Public Policy, The Pennsylvania State University, University Park, Pennsylvania, United States of America; Bigelow Laboratory for Ocean Sciences, UNITED STATES

## Abstract

Watersheds require collective care and management at local and regional levels to maintain their ecological health. The Chesapeake Bay’s last several decades of stagnantly poor ecological health presents a distinctive case study for explicating the challenges of motivating collective action across a diverse regional natural resource. Our study uses county- and individual-level descriptive analysis to examine interrelated framings of environmental quality, environmental sentiment, and political action at two critical moments in time—the 2016 and 2020 presidential elections. We find that demographic, environmental, and political characteristics vary with distance to the Chesapeake Bay and that linked environmental and political characteristics appeared to become more polarized between 2016 and 2020. We found no evidence that local environmental quality influenced new political actions such as voting; however, people already likely to vote were influenced by their pro-environmental values such as priorities around climate change.

## Introduction

Watersheds affect the life, well-being, and social interactions of people who live within their boundaries [1]. To maintain their ecological health, watersheds require collective care and management at local and regional levels. Despite their roles in organizing interactions and collective participation, social factors are often framed as contextual rather than central to the challenges of watershed management, with detrimental implications for environmental quality [1]. By discounting the importance of social factors and behaviors such as political participation, efforts towards watershed management may be less effective than they could be if such factors were considered.

A place of immense ecological and economic importance to the United States, the Chesapeake Bay Watershed (CBW) spans 44 million acres across 157 counties in 6 Mid-Atlantic states and the District of Columbia and is home to 23 million people [2]. The CBW suffers from poor ecological health due to high levels of nitrogen and phosphorous—mostly from agricultural runoff—and ineffective management processes [3,4]. Despite over three decades of organization and policy effort, water quality in the watershed has shown little improvement [5]. To meaningfully affect change, substantial collective action is needed among the watershed’s diverse stakeholders and inhabitants. Accordingly, understanding the socio-environmental features of the CBW is necessary to motivate efforts towards collective environmental management in the region. Co-management or adaptive management of watersheds foster locally sensitive processes that address complex multi-scale natural resources through shared information, goals, and power across boundaries, actors, and agencies [6–9]. A sense of collective identity is critical in these approaches to prevent the loss of a vital natural resource [8]. Yet, Cui et al. (2022) suggest that the vast size and heterogenous nature of the CBW may limit the capacity for such a collective identity to emerge, potentially explaining the lack of progress on environmental quality in the watershed [10]. For example, counties located near the Chesapeake Bay are most likely to be impacted by its environmental conditions, whereas counties far from the Bay may not be aware that they share a common identity as residents of the watershed, let alone understand how their actions affect those downstream.

In this study we examine the spatial and social characteristics of the CBW. We focus on the ways perceived and experienced environmental conditions relate to efforts towards collective action, measured through political action. We further investigate the spatial heterogeneity in local environmental quality and political participation throughout the CBW. The watershed is home to a diverse population in terms of political, social, and demographic characteristics. Further, it has long contended with environmental quality challenges, a fact that appears to defy well-documented relationships between environmental conditions, environmental attitudes, and political actions. People affected by either acute events (i.e., environmental disasters) or long-standing conditions (i.e., water and air pollution) are often motivated to engage in political actions in ways that reflect their experiences [11,12]. Taking this to the CBW, for example, people who live closest to the Bay or in areas with poor watershed quality would participate in collective efforts to improve their local conditions. Yet, whether or not such patterns arise throughout the CBW remains an empirical question. In fact, whether and how political behaviors align with environmental perceptions and experiences in the CBW has consequences for effective management and long-term environmental quality in the watershed.

## Background

Collective identity, which promotes collective action and management, does not emerge easily or rapidly. Developing a shared sense of understanding among people is a precursor to forming collective identity and promoting productive collaboration [8]. Conversely, when people do not agree on the problem, the underlying information, or the latent values associated with the concern, the resulting impasse, or “wicked problem”, results in a lack of progress [13].

Challenges related to environmental quality and management fit into this framework of “wicked problems”. Management of natural resources, such as watersheds, requires the development of local collective identities with shared priorities, values, and problem definitions. While such shared identity is needed, participants in collaborative processes are presumed to bring their own values and experiences to discussions [9]. Discussions among neighbors are traditionally seen as a way to resolve differences and incorporate diverse viewpoints toward co-management of natural resources [7]. However, the recent degradation of norms due to political polarization and social sorting suggests that such dialogues are less likely to promote or lead to a common foundation of understanding and values, thus making collective responses to environment challenges more tenuous and less effective. Rooted in these premises, three core ideas underpin our examination of environmental and political framings of the CBW: (i) political polarization has divided residents’ environmental values along social groupings; (ii) people are likely to behave in ways that reflect their social and physical contexts; and (iii) nationalization of political issues has divorced people from local contexts.

Since 1994, rapidly accelerating political polarization in the United States linked to people’s social identities has eroded the capacity for shared environmental sentiment across party lines [14–16]. During approximately this same period of ecological stagnation in the CBW, Democrats and Republicans have held increasingly negative views of each other resulting in disagreement over nearly every major issue [16]. Environmentalism is no exception. Since 1990, U.S. Senators’ environmental positions have become increasingly divisive along partisan lines [16]. This current state of division is rooted in the concurrent process of social sorting, or the alignment of social identities and political affiliation [14]. Even more concerning is the trend that more people disagree with an opposing party’s position than agree with their own party’s position [15]. This sentiment suggests that the current state of division is driven less by people coalescing around a shared set of values than a collection of individually held disagreements with the ideas of others. As a result, it has become difficult for people to separate their individually held environmental sentiments from their bond with a party affiliation that is galvanized by disagreement with the opposing party. Coupled with Papp’s (2022) axiom that “greener voters tend to vote for greener parties”, the last several decades have set a stage for substantial personal conflict around environmental issues [12].

People’s behaviors are a result of interactions between their individually held values and beliefs and the social, physical, and natural environments in which they spend time [12,17–20]. A person’s identity and values are traditionally rooted in place [21–25] through their unique social and cultural perceptions [21] and how they choose to spend time in a place [23,26]. People may form strong identities with a place or come to depend on the physical, psychological, or sociological resources of a place [25]. As a result, people are expected to act in ways that support that connection to place. Yet, the ways in which they express these actions may vary based on perceptions and ideologies.

While values and identities are traditionally rooted in place, the recent nationalization of politics in the United States has decoupled people’s political sentiments from their local bonds [27]. Where gubernatorial candidates once derived their campaign promises and motivation to seek compromises from local conditions and constituents, they now look to presidential and other national signals for guidance [27]. As a result, national- or state-level differences in political values that are tightly linked to individual identities are likely to influence people’s political participation in ways that diverge from local, place-based conditions. This may help to explain why people’s political behaviors often appear at odds with their personal interests and the priorities—such as environmental priorities—of the places in which they work and live. Such processes may be especially apparent in the CBW given its megaregional size and the inconsistency of how people learn about the Bay through local media markets [10]. That is, it becomes difficult to develop a shared CBW identity and national political trends discourage such collective identity building.

### Current conditions in the Chesapeake Bay watershed

The environmental and political sentiments and identities of people in the CBW are important factors for predicting the future successes or failures of watershed management efforts [28]. These characteristics, however, are not stagnant. The recent 2016 and 2020 presidential elections were periods of exemplary changes in people’s political, social, and environmental framings. While the historically high voter turnout in the 2020 election may be seen as a referendum on the then-incumbent president [29], it is also evidence of action taken by millions of people that reflects changes in values, identities, and perceptions over time.

**Fig 1:**
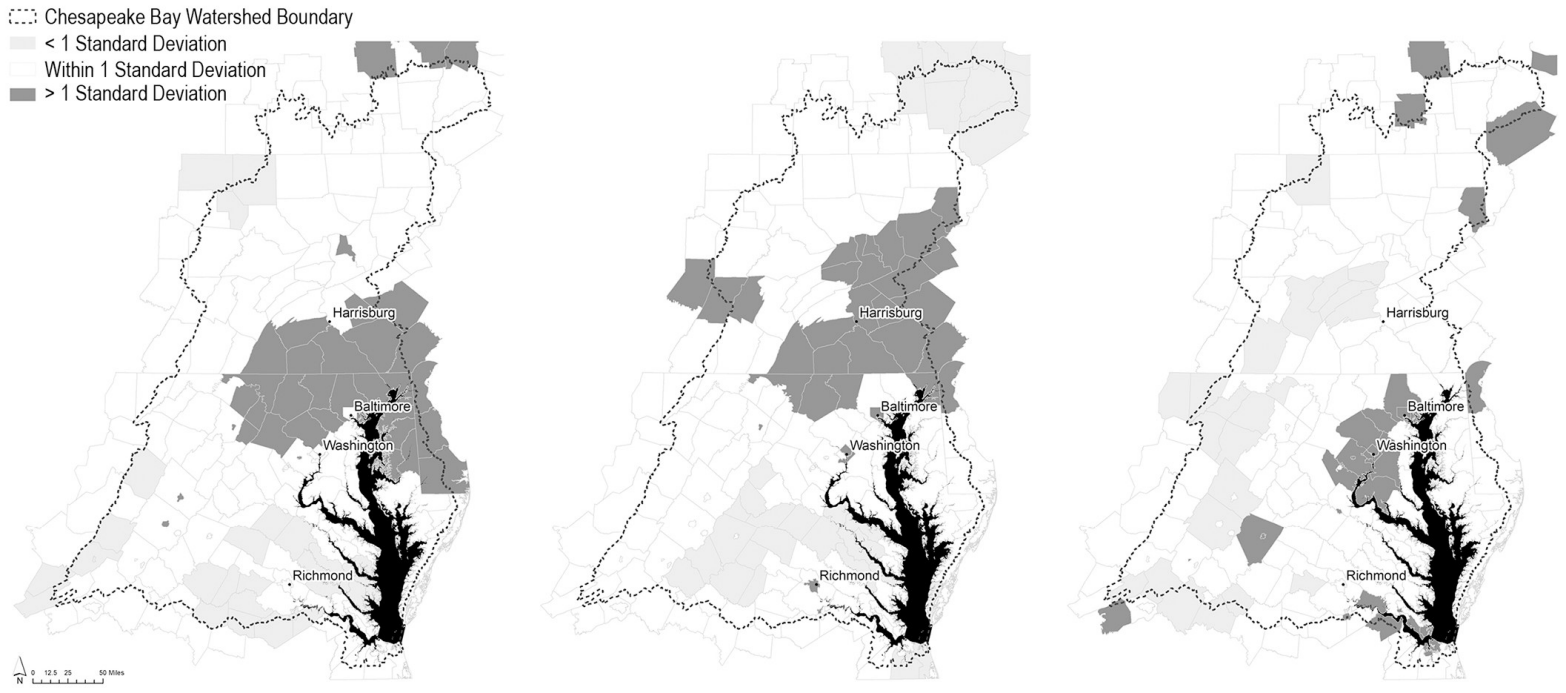
Maps of the Chesapeake Bay Watershed depicting significant county-level water quality (Panel A), air quality (Panel B), and percent of votes for the Democratic presidential candidate in the 2020 presidential election (Panel C). Maps generated by the authors using ArcMap v.10.7 with open access data from National Historical Geographic Information System (NHGIS), Shen et al. (2019), Hammer et al. (2020), van Donkelaar et al. (2019), MIT Election Data and Science Lab, (2018) [2,30–33].

To situate and describe the current conditions of the CBW, we generated a series of maps depicting environmental (air and water) quality and political sentiment characteristics in the region (Fig 1). These maps plot aggregated standard deviations of mean nitrogen in waterways [30], fine particulates in the air [31,32], and the percent of votes for the Democratic presidential candidate in the 2020 presidential election [33]. Water quality, air quality, and voting are spatially clustered in meaningful ways that reflect the coupled human and natural dynamics of the region. We tested the spatial autocorrelation of the data within each map, which confirmed there is a less than one percent likelihood these patterns result from random chance. All tests were performed using ArcMap 10.7’s *Spatial Autocorrelation (Morans I)* tool. The z-scores of the mapped variables are as follows: mean NO_3_ z-score = 13.44; mean PM2.5 z-score = 10.57; percent votes for Democratic candidate z-score = 12.31.

The resulting maps illustrate spatial differences across the CBW, especially highlighting differences between counties near and far from the Bay. Counties directly adjacent to the northern tributaries of the Chesapeake Bay and surrounding Harrisburg, Pennsylvania share generally poor water and air quality (Fig 1A and 1B, respectively), a condition that is likely indicative of the Bay’s persistently poor ecological health. In contrast, counties farther from the Bay in all directions—including in New York, Virginia, and West Virginia—share comparatively good water and air quality. Despite literature to the contrary, we observe little overlap between counties with poor environmental quality and high percentages of votes for the Democratic candidate, especially among counties with poor water quality. Generally, voting appears to follow traditional urban and rural patterns, with notable exceptions around Harrisburg, Pennsylvania, Richmond, Virginia, and in several urban-adjacent counties along the Bay’s coastline.

From our initial map-based description of the CBW, it seems likely that the people in counties with the poorest environmental quality do not connect their local environmental conditions with their political actions in a tactile or conceptual way. However, we cannot observe from the existing spatial conditions to what degree these characteristics interact spatially or temporally. To that end, in the remainder of the paper we examine: i) spatial differences in environmental and political characteristics of the CBW; ii) changes in environmental and political characteristics between the 2016 and 2020 elections; and iii) environmental and political interactions during this period.

We use county-level data from a variety of publicly available sources including the National Historical Geographic Information System (NHGIS) and individual-level data from the American National Elections Survey (ANES) to examine how spatial characteristics—including place in the CBW and environmental conditions—affect the ways in which people engage in political action. Using regression analysis, we assess the relationships between political action and environmental perceptions, environmental quality, and location within the CBW. These analyses provide insight into the extent to which perceived and realized environmental quality affect political behavior, with important implications for building collective identity and promoting collective action in a diverse region such as the CBW. Our descriptive results show that there are place-based differences in demographic characteristics and environmental quality throughout the CBW, which may have implications for patterns of environmental perceptions and political engagement in the region. Our regression results reveal that types of political behavior may adjust to environmental quality changes, yet the amount of political engagement does not appear to similarly change.

Our analysis makes two main contributions to the existing literature. First, it investigates a framework of political and environmental interactions in a critical regional watershed that has experienced decades of stagnant ecological health, with little improvement. Accordingly, our analysis provides suggestive evidence into the interactions between these framings and the ways in which environmental experiences do and do not stimulate political engagement. Second, these lessons have important policy implications. By understanding how the ways that environmental quality and environmental perceptions do and do not relate to political action, we can better conceptualize ways of developing collective identities in areas, such as the CBW, in which such identities are necessary to promote effective management and improve ecological health.

## Materials and methods

### Ethics statement

This study obtained ethical approval for existing data use through the Pennsylvania State University IRB (STUDY00018743). No primary data were collected for this analysis; as such, the authors of this study were not involved in the informed consent process.

### Data

The data for this study come from several different sources; we describe each in turn. County level data, including information on gender, race and ethnicity, educational attainment, and poverty levels, come from the National Historical Geographic Information System (NHGIS) [2]. These demographic and geographic data, identified at the county-level using Federal Information Processing Standard (FIPS) codes, are available for both 2016 and 2020. The most recent classifications (completed in 2013) of the rural-urban continuum codes (RUCC) were acquired from the United States Department of Agriculture (2024) [34]. For mapping purposes, and to identify proximity between counties in the Chesapeake Bay Watershed and the Chesapeake Bay itself, we merged the county-level data into a county-level shapefile [35]. All geographically referenced data was cleaned and analyzed using ArcMap v.10.7.

We use modeled estimates of county-level air and water quality. To measure air quality, we obtained annual, satellite-derived fine particulate (PM2.5) concentrations from Hammer et al. (2020) and van Donkelaar et al. (2019) [31,32]. These gridded satellite data are available at a 0.01x0.01 decimal degree grid (approximate 1.1 square kilometers (km2)). Using the same shapefiles described above, we processed the gridded satellite data using R into annual county averages by taking the average PM2.5 concentration across all grids within county boundaries. To measure water quality, we obtained seasonal estimates of total nitrogen (TN) and total phosphorous (TP) from Shen et al. (2019) [30]. Using data from 1994–2018, these data provide modeled estimates of freshwater TN and TP across the contiguous United States at a 1 km resolution. We used average TN and TP across all four annual seasons—spring, summer, fall, and winter. From the modeled data, we calculated annual county-level averages by averaging all 1 km segments of data that fell within county boundaries.

At the individual-level, we use data from the American National Election Studies (ANES). The ANES is a household survey that collects information about individual political actions, perceptions, and beliefs. For this study, we used the 2016 and 2020 ANES time series surveys. While much of this data is publicly available, we obtained the restricted county-level identifiers to facilitate merging county-level characteristics—including demographic and environmental variables—into the individual survey data. (All county-level, publicly available data is included as a supplement to this article in S1 Dataset. The authors are unable to share the restricted ANES data and direct interested users to https://electionstudies.org/data-center/restricted-data-access/).

We restricted the ANES sample to counties in the CBW, yielding an individual sample of approximately 200 individuals in 2016 and 400 individuals in 2020. (The post-election time series sample size increased from 3,648 to 7,440 between the 2016 and 2020 waves, leading to a larger CBW sample size for the 2020 analyses). From these data, we focused on constructs related to political behavior and environmental perceptions. While the ANES is a nationally representative survey, it is not representative at regional levels; thus, restricting our data to the CBW region limits the representativeness of our individual-level analysis. Accordingly, we consider our individual-level analysis as providing suggestive insight into the relationships between environmental experiences and perceptions and political action within the CBW.

From the ANES, we examined the following variables. First, to measure environmental perceptions, we used an indicator that took a value of one if the respondent supported increasing government spending for environmental priorities and zero otherwise (available in both the 2016 and 2020 time series) as well as an ordinal variable on strength of beliefs that climate change is affecting the weather ranging from 1 (low impact) to 5 (high impact) (available in only the 2020 time series). Second, we used four constructs of political participation. We used binary indicators for (i) voted—which took a value of one if the individual voted in the presidential election; (ii) voted Democrat—which took a value of one if the individual voted for the Democratic candidate in the presidential election; and (iii) voted Republican—which took a value of one if the individual voted for the Republican candidate in the presidential election. As the fourth construct, we used principal component analysis (PCA) to construct an index of political involvement that took into account whether an individual advertised for a candidate, went to candidate events, worked or volunteered for a campaign, contributed to a political candidate, and voted. We used the first component of this PCA analysis as our composite measure of political involvement.

### Methods

Our quantitative analysis examines how demographics, environmental quality, and political perceptions vary across the CBW. Our sample of the CBW contains 157 counties; following the definition from the Chesapeake Bay Foundation, all counties with at least a majority of the county area within the CBW watershed are included in our sample. We examine these relationships using simple means comparisons, comparing characteristics and perceptions of counties and individuals living adjacent to the Chesapeake Bay and living further away from the Chesapeake Bay. We define counties as Chesapeake Bay adjacent (n = 59) if part of the county boundary is located on the Chesapeake Bay coastline; those counties that do not share a border with the Chesapeake Bay (n = 98) are considered non-adjacent counties. For each comparison, we report the mean for each subsample (adjacent and non-adjacent) as well as the p-value on their difference from t-tests. We conduct comparisons at both the county and individual levels. At the county level, we compare differences in demographic characteristics and environmental quality between adjacent and non-adjacent counties in both 2016 and 2020. At the individual level, we compare differences in political behaviors as well as environmental beliefs and perceptions.

We also examine the correlates of individual political behavior using linear regression models, the results of which we report in the subsequent section. This allows us to examine political-environmental relationships, specifically examining if environmental conditions and perceptions impact individual political action. We estimate the following linear regression model separately for our samples from 2016 and 2020:

$$y_{ij}=\alpha +\beta_{1}env_{i}+\beta_{2}pm_{j}+\beta_{3}tn_{j}+\beta_{4}tp_{j}+\beta_{5}adjacent_{j}+\rho X_{j}+\varepsilon_{ij}$$ (1)

Our outcome of interest measures political behavior of individual *i* in county *j*. We consider four measures of political behavior. The first is a composite measure of political behavior constructed using principal component analysis (PCA). In addition to this composite measure, we consider three voting outcomes: (i) voting in the last presidential election; (ii) voting for the Republican candidate in the last presidential election; and (iii) voting for the Democratic candidate in the last presidential election.

The variable *env*_*i*_ measures individual perceptions of the environment—either a belief that the government should increase spending for environmental issues or a belief that climate change is having a large impact, depending on the specification; the variable *pm*_*j*_ measures annual average PM_2.5_ (in *μ*g/m^3^) in county *j*; the variables *tn*_*j*_ and *tp*_*j*_ are indicators that average annual total nitrogen and total phosphorous were at least one standard deviation above the mean values for the entire CBW; and *adjacent*_*j*_ is an indicator for living in a county adjacent to the Chesapeake Bay. In addition, we control for county-level demographics including race, ethnicity, income, education, and RUCC code (*X*_*j*_). We report robust standard errors.

The *β* coefficients estimate the correlations between environmental perceptions and conditions and Chesapeake Bay adjacency and individual political behaviors. Accordingly, these regressions allow us to examine how lived experiences across the CBW relate to political behavior—demonstrating how and if environmental conditions, and how people react to them, play a role in shaping political outcomes. Moreover, by examining these relationships separately for 2016 and 2020, we speak to these correlations at a time of heightened and increasing political polarization in the United States [14–16], offering insight into patterns of political behavior in this crucial Mid-Atlantic region. Importantly, we draw no causal claims from this analysis; rather, we find descriptive evidence regarding the relationships observed across the CBW.

## Results

The CBW is a complex heterogeneous landscape that is slowly changing over time. Our analysis of differences in county-level characteristics among counties adjacent to the Bay itself and those further from (non-adjacent to) the Bay using simple t-tests confirms the initial map-based descriptions that the spatial relationship of water quality, air quality, and demographic characteristics vary with respect to physical location in the CBW (see Table 1, Panel A). At the county-level, water quality, as measured by total nitrogen and phosphorous, is worse in counties closer to the Bay, although the levels of annual modeled nitrogen and phosphorous found throughout the CBW do not represent extremely high levels when compared to water quality standards. We also observed differences in air quality between adjacent and non-adjacent counties in both 2016 (6.67 and 6.87, p<0.10, respectively) and 2020 (6.01 and 6.15, p <0.10, respectively), which suggests slightly lower levels of fine particulates in adjacent counties during our study period. Counties also vary in demographics by proximity to the Bay. In counties adjacent to the Bay the population was approximately 20 percentage points more non-white in 2016 (p<0.001) and 22 percentage points more non-white in 2020 (p<0.001) than non-adjacent counties. While much less substantial in magnitude and less precise, adjacent counties were home to more Hispanic people than non-adjacent counties in 2016 (p<0.10) and 2020 (p<0.10). In 2016, counties adjacent to the Bay had higher levels of college education (p<0.05) and were home to slightly more female residents (p-value <0.001). Adjacent counties held more Bachelors degrees in 2016 (p-value <0.05), a traditional difference among urban and rural counties. Notably, we did not observe another traditional difference—in poverty levels—between adjacent and non-adjacent counties.

**Table 1:** T-tests for means comparisons between Bay adjacent and Bay non-adjacent locations.

	2016			2020		
	Adj	Non-adj	p-value	Adj	Non-adj	p-value
*Panel A*: *County-level comparisons*						
Total nitrogen	--	--	--	1.40	1.20	0.00
Total phosphorus	--	--	--	0.11	0.07	0.00
PM_2.5_	6.67	6.87	0.060	6.01	6.15	0.09
Female (%)	51.0%	50.0%	0.002	--	--	--
Non-white (%)	33.0%	13.0%	0.000	40.0%	18.0%	0.00
Hispanic (%)	6.0%	5.0%	0.060	8.0%	6.0%	0.08
Bachelors degree (%)	31.0%	25.0%	0.002	--	--	--
Poverty (%)	12.0%	13.0%	0.300	--	--	--
*Panel B*: *Individual-level comparisons*						
FT Democrats	55.1	42.4	0.010	67.0	52.7	0.00
FT Republics	35.2	47.8	0.010	24.5	39.7	0.00
Voted (%)	91.0%	90.0%	0.900	89.0%	86.0%	0.20
Voted Democratic (%)	45.0%	31.0%	0.030	56.0%	35.0%	0.00
Voted Republican (%)	21.0%	37.0%	0.010	16.0%	32.0%	0.00
Increase env spending (%)	65.0%	49.0%	0.010	70.0%	63.0%	0.09
Decrease env spending (%)	11.0%	11.0%	0.900	4.0%	6.0%	0.20
Climate change effects	--	--	--	3.91	3.56	0.003
Climate change importance	--	--	--	3.71	3.35	0.003

Table notes: In Panel A, all results from t-tests assessing differences in means between counties adjacent to the Bay (adj) and non-adjacent to the Bay (non-adj) in 2016 and 2020. Results reported as mean for each group and p-value on the difference. Total nitrogen and total phosphorus (measured in ppb) come from modeled water quality data and are available only for the combined period of 1994–2018; we report this value only for 2020. In Panel B, all results from t-tests assessing differences in means between individuals living in counties adjacent to the Bay (adj) and non-adjacent to the Bay (non-adj) in 2016 and 2020. Results reported as mean for each group and p-value on the difference. FT stands for “Feelings Thermometer”, which ranges from 1 (cool or very negative feelings towards item) to 100 (hot or very positive feelings towards item). Increase and decrease environmental spending are indicators that take a value of one for individuals who believe environmental spending should increase or decrease, respectively. Voted, voted Democrat, and voted Republican are indicator variables that take a value of one for individuals who voted, voted Democrat, and voted Republican in the presidential election, respectively. Climate change effects is an ordinal variable ranging from 1 (no impact) to 5 (high impact), available only in 2020. Climate change importance is an ordinal variable ranging from 1 (low importance) to 5 (high importance), available only in 2020. Significant differences (p<0.1) are marked by darker text; insignificant differences (p>0.1) are marked by lighter text.

We also examine how environmental perceptions and political actions vary among individuals across the CBW, again differentiating groupings by proximity to the Bay and again estimating these differences separately for 2016 and 2020. At the individual level, we observe that environmental and political sentiment appears to have shifted both temporally and spatially between 2016 and 2020 (Table 1, Panel B). Our results indicate a broad shift toward pro-environmental and pro-Democratic support across the study area.

Respondents who live closer to the Bay are generally more liberal leaning than those living in non-adjacent counties. This characteristic appeared to become more polarized between 2016 and 2020. Respondents in adjacent counties reported higher support for Democrats in 2016 by 12.7 percentage points (p <0.01), which increased to 14.3 percentage points (p<0.001) in 2020. Likewise support for Republicans in 2016 was higher in non-adjacent counties by 12.6 percentage points (p<0.01), increasing to 15.2 percentage points (p-value <0.001) in 2020. Further, respondents were more likely to vote for the Democratic Presidential candidate in adjacent counties by a difference of 14 percentage points (p<0.05) in 2016, which increased to 21 percentage points (p<0.001) in 2020. Respondents in non-adjacent counties, on the other hand, were more likely to vote for the Republican Presidential candidate in 2016 (p<0.01) and 2020 (p<0.001), the difference did not increase between election years.

Broadly, people in counties closer to the Bay demonstrate more environmental-friendly perceptions. Respondents adjacent to the Bay reported higher support for increased environmental spending in 2016 by a difference of 16 percentage points (p<0.01) than non-adjacent counties, though the difference decreased to 7 percentage points (p < .10) in 2020. This decrease, however, was at least partially driven by higher beliefs in both areas that environmental spending should increase. Respondents in adjacent counties also felt climate change was of greater importance as a policy issue than those residing in non-adjacent counties (3.71 and 3.35, respectively, p<0.05). Comparisons of means between adjacent and non-adjacent counties provide some insight into the spatial patterns in political leanings and environmental sentiment throughout the CBW, yet they are unable to account for more nuance in these relationships. For this, we turn to multivariate linear regression models, which examine the correlates of political behavior throughout the CBW.

### Regression results

Our linear regression models describe how variation in political involvement and voting behavior can be explained by determinants such as environmental spending preferences, air quality, water quality, and adjacency to the Bay. Despite the emergence of some interesting patterns, we note that the majority of our estimates across both years are imprecisely measured (Table 2). However, three notable patterns arise in our results.

**Table 2:** Linear regression results.

	Political involvement	Voted	Voted Republican	Voted Democrat
*Panel A*: *2016 sample*				
**More env. spending**	-0.25	0.01	-0.29***	0.34***
	(0.23)	(0.05)	(0.06)	(0.07)
**PM**_**2.5**_	-0.10	0.05	0.01	0.04
	(0.23)	(0.04)	(0.06)	(0.05)
**High total nitrogen**	-0.02	0.01	0.35***	-0.36***
	(0.34)	(0.08)	(0.12)	(0.11)
**High total phosphorus**	0.83**	0.09*	-0.12**	0.17**
	(0.35)	(0.05)	(0.05)	(0.09)
**Adjacent to bay**	-0.12	0.05	-0.05	0.05
	(0.17)	(0.04)	(0.05)	(0.06)
Observations	197	197	232	232
R^2^	0.14	0.07	0.27	0.25
*Panel B*: *2020 sample*				
**More env. spending**	0.20	0.04	-0.35***	0.37***
	(0.12)	(0.04)	(0.04)	(0.04)
**PM**_**2.5**_	0.17	0.02	-0.06	-0.001
	(0.15)	(0.04)	(0.04)	(0.05)
**High total nitrogen**	0.20	-0.01	0.08	-0.14*
	(0.27)	(0.06)	(0.08)	(0.08)
**High total phosphorus**	-0.06	-0.0001	-0.03	0.04
	(0.20)	(0.04)	(0.04)	(0.04)
**Adjacent to bay**	0.08	0.07**	-0.04	0.10**
	(0.15)	(0.03)	(0.04)	(0.04)
Observations	390	390	459	459
R ^2^	0.10	0.11	0.26	0.23

*Table notes*: Coefficient estimations from linear (OLS) regression. Results reported as coefficient (robust standard error). All regressions control for black, Hispanic, income, high school diploma, and county RUCC code.

*p<0.1;

**p<0.05;

***p<0.01.

First, both experienced environmental quality and perceptions about environmental policy priorities have more meaningful relationships with how individuals engaged in politics—that is, for which candidate they voted—rather than if individuals participated in politics by voting. Across our analyses for both 2016 and 2020, we find that voters who prioritized increases in environmental spending were more likely to vote for the Democratic presidential candidate and less likely to vote for the Republican presidential candidate. These environmental perceptions, however, were not related to whether an individual chose to be involved in the political process via voting or other political behaviors such as contributing to campaigns or attending political events.

Second, measures of water quality have a stronger relationship with political behavior in 2016 compared to 2020, with potentially interesting implications for watershed management. We find that voters in counties with elevated phosphorus levels were more engaged in the political process and more likely to vote for the Democratic presidential candidate in 2016. This suggests that local water quality may have been a motivating factor for political engagement in our first study period. By 2020, however, we do not see the same trend. Rather, we find that water quality metrics are not related to political involvement or to voting behavior. This could be a result of the increasing political polarization between 2016 and 2020, which, perhaps, led to a divergence between local environmental quality concerns and national voting behavior.

Third, there appear to be spatial patterns of political engagement based on adjacency to the Bay; these results are driven by our 2020 sample. We see that, in 2020, residents in Bay-adjacent counties were more likely to vote and more likely to vote for the Democratic presidential candidate. While we cannot disentangle differing mechanisms for this relationship—such as demographics, environmental quality, or other local political priorities—the observed relationship suggests that spatial differences are important for understanding voting patterns in the CBW.

Using our 2020 data, we are also able to examine the relationships between climate change beliefs and political engagement (Table 3) using data from new questions added to the ANES 2020 time series survey. These results largely mirror our findings related to environmental spending priorities, suggesting considerable overlap between individuals who prioritize environmental spending and voters who prioritize climate policy. We find that individuals who cite climate as an important policy priority are more likely to be involved in a broad set of political participation behaviors, more likely to vote Democratic, and less likely to vote Republican compared to those who do not cite climate as an important policy priority. We also see the same patterns with respect to the relationship of Bay-adjacency. Surprisingly, this pattern does not appear to extend specifically to voting behaviors. Notably, these observations generally align with long-standing urban-rural characterizations in the region, which we presume accounts for a substantial degree of explanation, although our models do control for such county-level factors to the extent possible.

**Table 3:** Climate change regressions with 2020 sample.

	Political involvement	Voted	Voted Republican	Voted Democrat
**High climate impact**	0.12**	0.006	-0.17**	0.17**
	(0.06)	(0.01)	(0.02)	(0.02)
**PM**_**2.5**_	0.15	0.01	-0.03	0.01
	(0.15)	(0.04)	(0.05)	(0.05)
**High total nitrogen**	0.26	0.02	0.05	-0.12
	(0.25)	(0.06)	(0.07)	(0.07)
**High total phosphorus**	-0.05	0.02	-0.02	0.05
	(0.20)	(0.04)	(0.04)	(0.06)
**Adjacent to bay**	0.09	0.08**	-0.04	0.11**
	(0.15)	(0.03)	(0.04)	(0.04)
Observations	388	388	414	414
R^2^	0.10	0.10	0.35	0.31

*Table notes*: Coefficient estimations from linear (OLS) regression. Results reported as coefficient (robust standard error). All regressions control for black, Hispanic income, high school diploma, and county RUCC code.

*p<0.1;

**p<0.05;

***p<0.01.

Our results suggest that the ecological health of the Bay covaries with political decisions (such as who to vote for) but not necessarily with political participation (whether or not to vote). Thus, as the ecological health in the Bay changes, we may expect to also see shifts in political decisions. While the ecological health of the Chesapeake Bay has been consistently poor, individual measurements do vary somewhat year to year. For example, the University of Maryland’s Center for Environmental Studies (2022) EcoReport Card of the Bay reports that the nitrogen score fell to its lowest level between 2016 and 2019 before rebounding to its highest reported score in 2020 [5]. In contrast, the phosphorus score has consistently fallen from 2016 to 2020. These conditions demonstrate that the CBW is a landscape in constant flux, with implications for political engagement, the development of collective identity, and the potential for policy-directed improvements to environmental quality. Our results illustrate spatial congruency between political-environmental dimensions of the CBW, which may align with political engagement mirroring national, rather than local, trends. However, in our analysis we explore only a limited set of theoretically representative, but not a holistically exhaustive, demographic, environmental, and political variables. As such, identifying causal environmental impacts on voting behavior and other political engagement are beyond the scope of our current data and analysis and analysis.

## Discussion

The CBW is a complex, heterogenous region in which environmental and political patterns are not easily described nor static for extended periods of time. Yet, despite its dynamics, some patterns emerge from our descriptive analysis. Broadly, these patterns and trends appear to align with the assumption that people’s environmental values and exposure to environmental conditions are aligned with their political actions [12]—albeit in nuanced ways. First, there are observable differences in the environmental quality, sociodemographic characteristics, and political sentiments of people and places across the CBW. Broadly, counties near the Bay have poorer water and air quality and are home to more racially diverse, educated, and Democratic-leaning populations. Second, neither environmental quality nor proximity to the Bay appeared to consistently correlate with political participation, either broadly or specifically in voting. However, environmental quality, values regarding climate change and environmental spending, and proximity to the Bay does significantly correlate to people’s voting decisions. Third, many of these patterns, especially Democratic sentiments, appeared to grow stronger between 2016 and 2020. We find evidence that political polarization is increasing across both space and time in the CBW in ways that align with changes in the local environmental quality and people’s engagement in political actions, as noted in the differences observed between our analyses for 2016 and 2020. This shift appears to be occurring despite little change in the demographic characteristics of counties adjacent to the Bay that traditionally lean toward supporting the environment—namely, female, non-white, Hispanic (classically underrepresented groups in voting), and higher educated populations [36,37]. Extrapolating from these findings, our results suggest that experienced environmental conditions, particularly when detached temporally or spatially from adverse consequences (such as the long-term effects of poor water quality), may be insufficient to motivate collective action around watershed management. Rather, intervention to motivate a shared ecological identity may be needed to improve outcomes in vulnerable ecosystems, such as the CBW.

### Limitations

There are several factors that limit the scope of and findings from our analysis. First, with respect to the potential for ecological fallacy in our interpretate of our results, we acknowledge that our limited analytical precision could reflect the data’s inability to capture the vastness and complexity of the CBW region. Over its 44-million acres, the CBW is home to a wide variety of land covers, climate conditions, social community groups, and individuals. Simple patterns of independence between two measurements or consistent covariances are unlikely to explain much of the real variation in urban, suburban, and rural place types. Real places at this scale are diverse and messy. When studying such multi-county areas, it is not uncommon to observe significant, yet small, coefficient values. In the larger socio-ecological system, unique influences of biotic systems and human behavior are difficult to isolate and frequently share moderating covariates. As a result, we interpret our findings here within the context of large-scale socio-ecological systems that appear to be changing in alignment with each other. But further detailed efforts are necessary to confirm interrelationships among those systems.

Second, voters traditionally do not list the environment or their health as a top priority. For example, in a survey of top priories ahead of the 2018 Midterm Elections, voters expressed strong partisan divide but generally reported strong feelings about the economy, broadly, health care, immigration, and foreign policy [38]. Within these long-held expectations of voting trends, we were not surprised by seeing that neither the local environmental quality nor proximity to a natural resource as strong (i.e., high magnitude) determinants of political participations. However, a recent poll among voters of color revealed that climate change had risen in its importance as an important issue facing the country, alongside immigration, abortion, and gun violence. Notably, jobs, economic growth, and inflation remained dominant [39]. While the historic axioms of voting priorities appear to remain true, more specific groups of underrepresented voters are revealing environmental values-based priorities.

Finally, the data themselves—both at the county and individual levels—present limitations to the analysis. At the county level, there is some inconsistency in data availability between 2016 and 2020. Moreover, counties are quite large and diverse themselves, motivating individual analysis. The individual-level data, however, also present limitations. While representative at the national level, the subsample of CBW individuals in the ANES is not representative of the watershed. Accordingly, the analytical results should be viewed as providing only preliminary insights into relationships between environmental quality and sentiments and political actions. More work is needed to fully characterize these relationships and understand the causal links between environmental characteristics and political behavior.

## Conclusions

Effective watershed management relies on collective action via shared priorities, values, and common understandings. We suggest that the ephemeral nature of these spatiotemporal conditions is central to unraveling a persistent wicked environmental problem, both in the CBW and other regional-scale natural resources facing long-term management challenges. Polarization increasingly separates people into social groups with differing values with little opportunity for shared understanding. We see evidence of this process reflected in the 2016 and 2020 presidential election results as the gap between Democratic and Republican support in counties grew, especially county location grew farther from the Bay. Our findings that people’s decision to vote, or, more generally, engage in other forms of political participation, appears to change little in alignment with the local environmental quality is instructive in that it points to the tension between local and national political motivations. Further, our findings that environmental values toward climate change and level of participation suggest that environmental priorities appear to covary may become increasingly important for political behavior, even if national and global issues appear stronger motivators than do local environmental conditions.

Theoretically, we interpret these observations to mean that that people are not motivated beyond the actual or perceived barriers of their physical and/or social contexts to vote. However, people who were already likely to vote were influenced by their pro-environmental values. Balint et. al. (2011) state that acknowledging and sharing inherent values among stakeholders is a key step in addressing wicked environmental problems [13]. Likewise, as political engagement becomes increasingly values-based, we suggest that our findings support the need to decipher the array of values as a first step in encouraging collective actions and engagement in watershed management.

## Supporting information

S1 Dataset County-level dataset used for analysis generated from publicly available sources. (XLSX)
